# Do phages impact microbial dynamics, prokaryotic community structure and nutrient dynamics in Lake Bourget?

**DOI:** 10.1242/bio.013003

**Published:** 2015-10-23

**Authors:** Antony Meunier, Stéphan Jacquet

**Affiliations:** INRA, UMR CARRTEL, 75 avenue de Corzent, Thonon-les-Bains 74203, Cedex, France

**Keywords:** Viruses, Bacteria, Archaea, Richness, Nutrient, Regulation, Lake

## Abstract

Phages are the most abundant and diversified biological entities in aquatic ecosystems. Understanding their functional role requires laboratory experiments on a short time-scale. Using samples of surface waters of Lake Bourget, we studied whether viruses impact (i) the abundance patterns of the bacterial and phytoplankton communities, (ii) a part of the prokaryotic community composition (both for Eubacteria and Archaea), and (iii) the recycling of nutrients and/or organic matter. Three experiments were performed (one each in February, March and April) at the transition between winter and spring in 2013. The experiment reduced or increased the abundance of virus-like particles in samples containing only the picoplanktonic fraction. Viral and cellular abundances, bacterial and archaeal community structures as well as nutrient concentrations were analysed every 24 h for 3 days. Some of the results reveal that increasing the phage abundance increased the diversity of the eubacterial community. Consistent with the ‘killing the winner’ concept, viruses are thus likely to significantly change the composition of the bacterial community. This suggests a positive association between viral abundance and bacterial diversity. In contrast, the composition of the archaeal community did not seem to be affected by phage abundance, suggesting the absence of viral control on this community or the inability to observe it at this period of year, either based on the time scale of the investigation or because the archaeal virus titre was too low to induce a significant and visible effect. Lastly, we were unable to demonstrate viruses driving the cycling of nutrients or the response of plankton to nutrient concentration changes in a significant way, suggesting that the role of viruses may be subtle or difficult to assess through the use of such experimental procedures.

## Abstract (precis)

**Summary:** Phages can exert important control on the structure of the bacterial community. By contrast, neither the archaeal community nor the cycling of nutrients seems to be affected by phage abundance.

## INTRODUCTION

Viruses are found wherever life occurs, and current estimates (ca. 10^31^ viruses) suggest that they are the most abundant biological entities on the planet (Suttle, 2005). During the last two decades, these particles have been shown to play key roles in the functioning of aquatic ecosystems. As actors of cellular lysis, viruses can directly affect the abundance of planktonic cells as well as the structure of their communities (Wommack and Colwell, 2000; Weinbauer, 2004; Weinbauer and Rassoulzadegan, 2004; Suttle, 2007). The probability of contact between a host and virus, which depends on prey density, suggests that the most dominant species will be the most impacted. This hypothesis of regulation by viruses is at the origin of the ‘killing the winner’ (KtW) concept proposed by Thingstad and Lignell (1997) and Thingstad (2000). Apart from the control of algal blooms, the lytic cycle has been reported to be responsible for 10 to 50% of the daily loss of bacterial biomass, on average, which represents a loss of 10 to 20% of the bacterial production in aquatic ecosystems (Weinbauer, 2004; Jacquet et al.*,* 2010).

Viruses can influence the genetic diversity of prokaryotes in various ways. They can affect the community composition of prokaryotes by ‘killing the winner’ and keeping competitive dominants in check. This may sustain species richness and the amount of information encoded in genomes. Viruses can also transfer genes between species, generating genetic variability in prokaryotes, and thus, influencing speciation (Weinbauer and Rassoulzadegan, 2004; Suttle, 2005; Sime-Ngando and Colombet, 2009). Viral lysis leads to the release of cellular material that is made available for less competitive species, and in this way, it allows for the maintenance of prokaryotic diversity. Some experiments have demonstrated that viruses modify the composition of the host community by forcing the establishment of resistance mechanisms in infected cells (Middelboe et al., 2001; Thomas et al., 2012). Whether the apparent diversity results from the death of the dominant species (that are replaced by rarer species) and/or is an indirect consequence of viral lysis and nutrient turnover that fuel non-infected populations remains a question of debate. Some authors have suggested an effect of viruses on the composition of the marine bacterial community (e.g. Middelboe, 2000; Hewson and Fuhrman, 2006; Winter et al., 2004 or Sandaa et al., 2009), whereas other studies, performed on lakes, have not revealed any significant effect (e.g. Berdjeb et al., 2011 and references therein). However, the study of Jardillier et al. (2005) showed an effect of viral lysis on the composition of the prokaryotic community in eutrophic lakes, revealing that the effect of viral lysis remains ambiguous.

Cell lysis affects biogeochemical cycles (Brussaard et al., 2008). A fraction of the lost production is composed of carbon and nutrients, such as nitrogen and phosphorus, which are not transferred to higher trophic layers. Viruses are thus responsible for what is referred to as the “viral shunt” in the transfer of organic matter, increasing the dissolved and, in particular, the organic nutrient pool (Wilhelm and Suttle, 1999; Sime-Ngando and Colombet, 2009; Thomas et al., 2011) available to non-infected microorganisms. This release seems to have a weak impact on carbon cycling compared to the microbial loop (Azam and Worden, 2004). However, it has been estimated that primary producers supply between 6 and 26% of the fixed carbon in pelagic environments in the form of dissolved organic carbon, which is derived from cell lysis driven by viruses (Fuhrman, 1999; Wilhelm and Suttle, 1999). Experiments designed by Weinbauer et al. (2011) and Shelford et al. (2012) have suggested that the viral lysis of some marine bacteria can be the origin of the release of ammonium. This nitrogen remineralisation could maintain phytoplanktonic production, for example, of the picoalgae. More recently, Ankrah et al. (2014) showed that, following lysis, compounds such as glutamate and glutamine (which are preferred sources of nitrogen over ammonium in bacteria) were rapidly assimilated by non-infected metabolically active cells. This was done using LC-MS/MS based metabolomics to highlight the chemical composition of DOM following the lysis of a *Sulfitobacter* species by a roseophage. Weitz et al. (2015) designed a multitrophic model that included the effects of viruses on microbial food webs for the first time and showed that the presence of viruses (i) increases the recycling of organic matter, (ii) reduces transfer to higher trophic levels and (iii) increases net primary production. Finally, Dean et al. (2008) revealed that bacterial lysis by viruses in Lake Erie could release fairly large quantities of phosphorus each day, suggesting a major role in the recycling of phosphorus. Currently, there is no longer any doubt that the virus-induced influx of organic matter can enhance bacterial production and fuels the microbial loop and the cycling of inorganic nutrients (see Shelford et al., 2014 and references therein).

Studies conducted in fresh waters (typically large and deep lakes) are scarce, and many questions dealing with viral effects on the nutrient dynamics and community structure remain unanswered. Consequently, we manipulated the abundance of viruses in natural waters from Lake Bourget to assess the impact on (i) the abundances of the prokaryotic and phytoplanktonic communities, (ii) the structure of bacterial and archaeal communities and (iii) the dynamics of nutrients and organic matter recycling. Our working hypothesis was that increasing or decreasing the abundance of viruses would modify plankton and nutrient dynamics by changing the composition of the prokaryotic community.

## RESULTS

### Initial conditions at the onset of each experiment

**Fig. 1.**
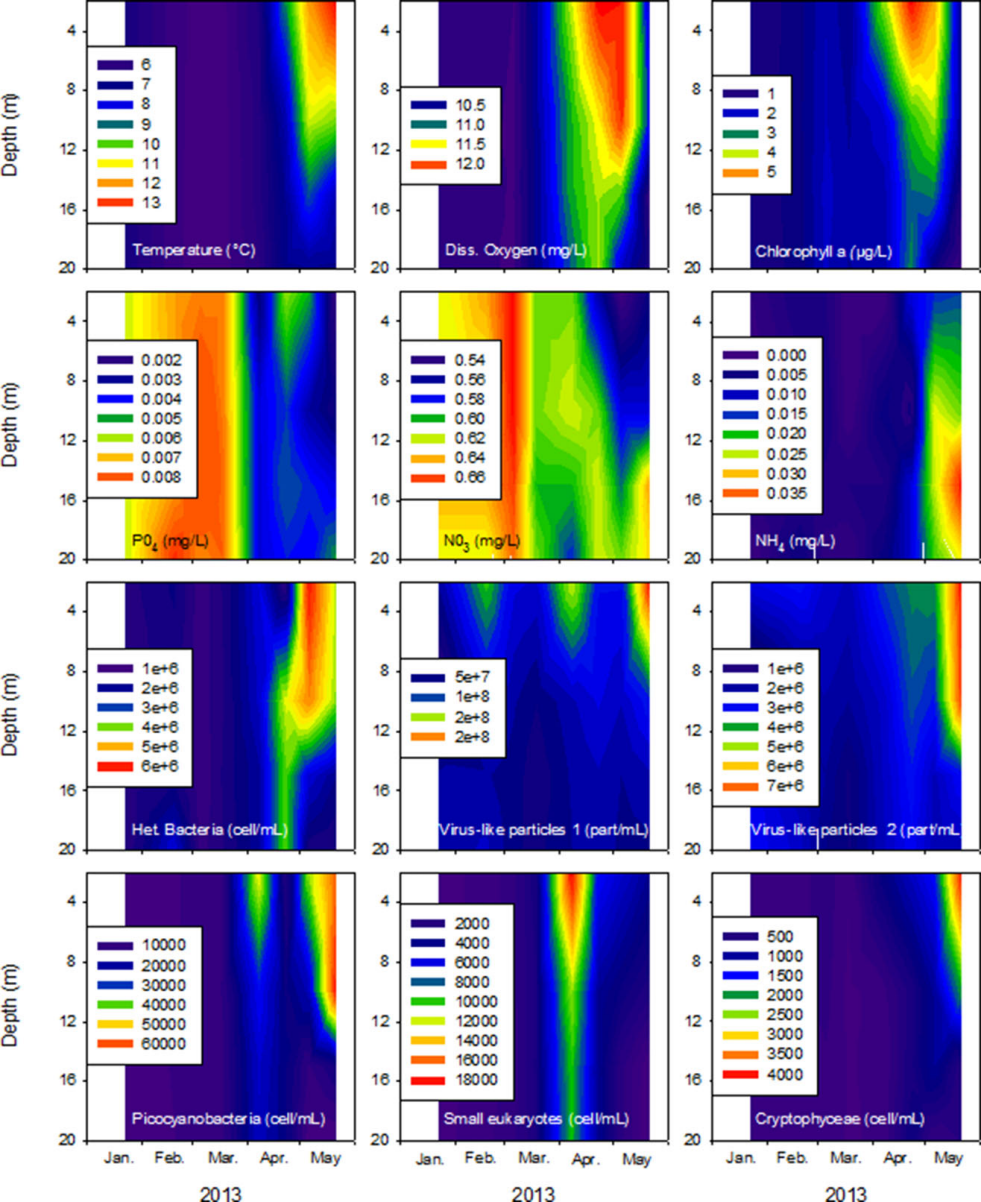
**Evolution of the main parameters between January and May 2013 in surface water (0-20 m) at the reference station of Lake Bourget**. Top line panels: temperature, dissolved oxygen and chlorophyll a concentration, second line panels: orthophosphate, nitrate and ammonium concentrations, third line panels: heterotrophic prokaryote and virus-like particle abundances, bottom line panels: picocyanobacteria, small eukaryotes without phycoerythrin and cryptophyceae abundances.

The experiment was repeated on three occasions, in February (M1), March (M2) and April (M3) 2013, when both the physical and chemical conditions were different and the biological compartments were highly dynamic (Fig. 1). The water temperature and dissolved oxygen and chlorophyll *a* concentrations were all significantly higher in April, while the concentrations of PO_4_ and NO_3_ were higher earlier in the year (*P*<0.05). The abundances of the different biological components tended to increase throughout the year, and the highest cellular or particle densities were observed in the top 10 m. The mean abundance of heterotrophic prokaryotes at the beginning of each experiment (i.e. corresponding to the sampling date) were 1.7×10^6^, 1.4×10^6^ and 3.2×10^6^ cells ml^−1^ in M1, M2 and M3, respectively. Two virus-like-particle (VLP) populations were detected and named VLP1 and VLP2, as previously defined (Personnic et al., 2009), and will be referenced accordingly in this manuscript. Their abundances at the start of the M1, M2 and M3 experiments were 7.5×10^7^, 4.4×10^7^, and 6.7×10^7^ particles ml^−1^ for VLP1 and 2.2×10^6^, 1.7×10^6^, and 3.2×10^6^ particles ml^−1^ for VLP2, respectively. For the autotrophic community, picocyanobacterial communities were the dominant group, with abundances increasing from 7.9×10^3^ (in February) to 9.9×10^3^ (in March) to 1.5×10^4^ cells ml^−1^ at the end of April. The second group was composed of cryptophytes, for which the numbers changed from 2.6×10^2^ (in February) to 1.9×10^2^ (in March) to 7.3×10^2^ cells ml^−1^ (in April). For the other small autotrophs grouped together, abundances evolved from 1.0×10^3^ (in February) to 2.5×10^3^ (in March) to 4.6×10^4^ cells ml^−1^ (in April).

### Effect of treatments on viral abundances

**Fig. 2.**
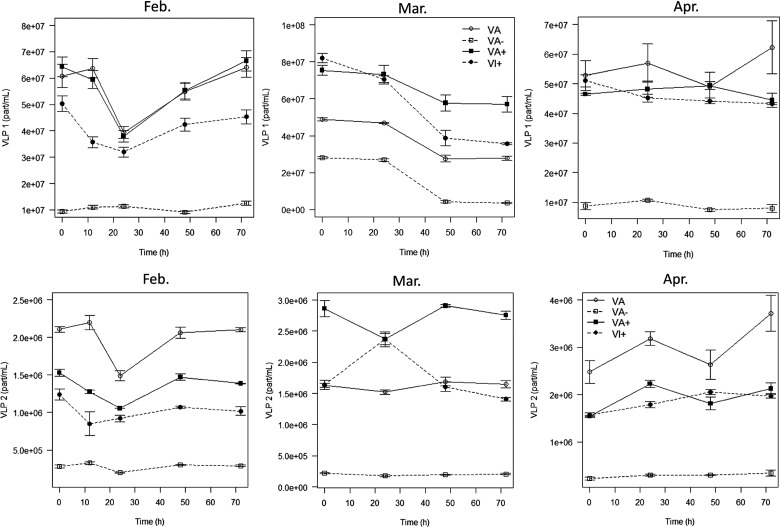
**Evolution of the abundances of the virus-like-particle group 1 and 2 at the three periods examined and in the different treatments.** The evolution of the viruses was observed using FCM at the three periods examined and on treatment with normal (VA), increased (VA+) or reduced (VA−) viral abundance, or with or virus-inhibited fractions (VI+). Data is represented as mean±s.e.m.

Our experimental design consisted of increasing or decreasing viral abundances compared to natural conditions (Fig. 2), which were referred to as VA (normal conditions), VA+ (increased abundance) and VA− (reduced abundance). Virus abundance was significantly enhanced in the VA+ treatment of experiment 2 (M2), which was conducted in March for both VLP1 and VLP2 (*P*<0.05). At t0, VLP1 was 1.5-fold higher in VA+ compared to VA and was 1.75 higher for VLP2 (*P*<0.05). No changes were observed, however, during both the M1 and M3 experiments. Comparatively, the expected decrease in the VLP (i.e. VLP1+VLP2) abundance was effective for the three experiments. At each period, the VA− condition was, as expected, approximately 6.5 and 7.5 times lower for VLP1 and VLP2, respectively, for M1; 1.7 and 7.4 times for VLP1 and VLP2, respectively, for M2; and 6.6 and 8.6 times for VLP1 and VLP2, respectively, for M3 (*P*<0.05).

Over the course of the experiment, the viral dynamics were very different between the different periods, while they were very similar between the treatments during the same period. Additionally, VLP1 and VLP2 behaved differently.

### Viral production and lysogeny

These parameters were measured only during the second experiment, M2. At t0, viral production was similar between treatments, with 1.37×10^5^±0.91×10^5^ particles ml^−1^ h^−1^. After 24 h, viral production decreased in the VA and VA− treatments (VP_VA_=3.61×10^3^ particles ml^−1^ h^−1^ and VP_VA−_=1.83×10^3^ particles ml^−1^ h^−1^), and no production was measured in both the VA+ and VI+ (virus-inhibited) treatments when considering all of the VLPs. However, we measured a small VLP2 production, i.e. 2.05×10^4^ particles ml^−1^ h^−1^ between t24 and t48 in VA+. The fraction of lysogenic bacterial cells (FLC), after 24 h, was 11.8% in VA, 100% in VA+ and VI+ and 0% for VA− (not shown).

### Effect of treatments on the abundance and growth rates of different microbial groups

**Fig. 3.**
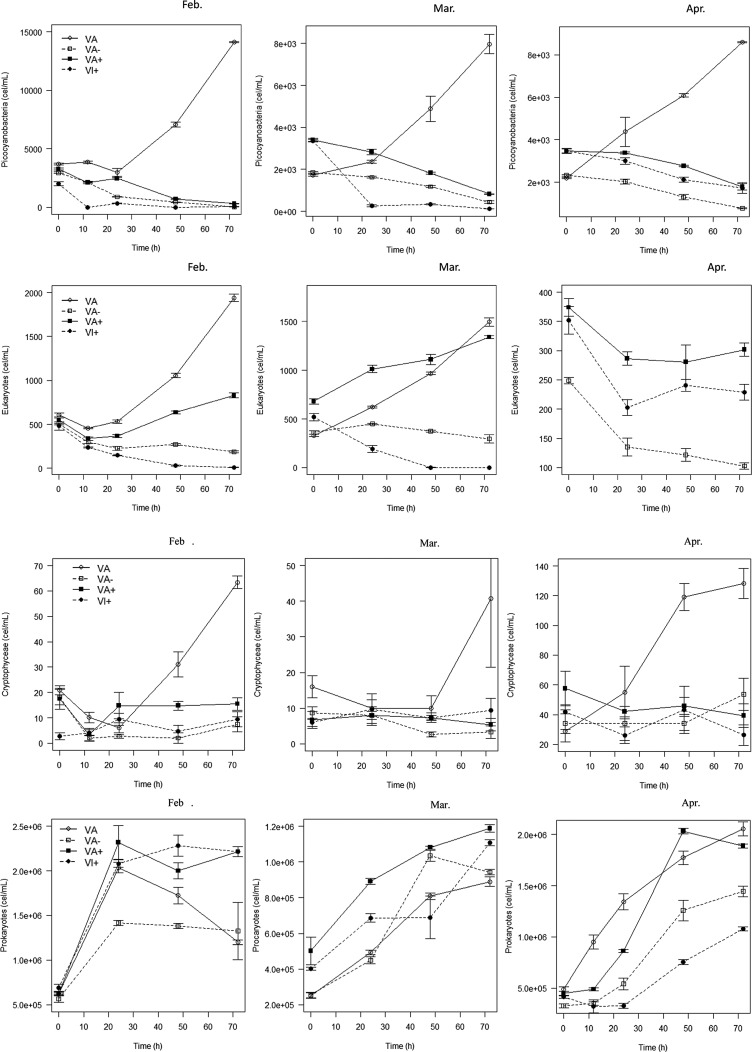
**Evolution of the abundances of the different autotrophic or heterotrophic populations in response to viral abundance.** The evolution of autotrophic or heterotrophic populations (i.e. the picocyanobacteria, the small eukaryotes without phycoerythrin, the cryptophyceae and the heterotrophic prokaryotes) were observed using FCM at the three periods examined and on treatment with normal (VA), increased (VA+) or reduced (VA−) viral abundance, or with or virus-inhibited fractions (VI+). Data is represented as mean±s.e.m.

We always observed the same trend for the picocyanobacterial community, with a marked increase in cell abundance in the VA treatment, while the concentrations decreased in the other treatments (Fig. 3). However, while it takes 24 h to observe this increase in M1, it was faster in M2 and M3. The highest growth rates of this community reached 0.86 day^−1^, 0.72 day^−1^ and 0.69 day^−1^ during M1, M2 and M3, respectively. We also observed that the decrease was less marked in the VA+ treatment compared to VA− and VI+.

As for the picocyanobacteria, the eukaryotes increased in the VA treatment in each period, with high growth rates for this mixed group. The maximum growth rates recorded in M1, M2 and M3 were 0.68, 0.63 and 0.61 day^−1^, respectively. However, in contrast to pycocyanobacterial communities, eukaryotes also increased in the VA+ treatment in the M1 and M2 experiments (but not in M3), but at a lower rate than in VA (i.e. with a maximum of 0.54 and 0.4 day^−1^). For both VI+ and VA−, the abundances decreased.

The patterns observed for the cryptophytes were different, with a net increase in cell abundance in the VA treatment after t24 during M1 or after t48 during M2, while the increase already began at t0 during M3. There were no marked differences between the other treatments, in which the group remained relatively constant (at *P*<0.05). The maximum growth rates reached 1.6, 1.4 and 0.47 day^−1^ in M1, M2 and M3, respectively.

Lastly, we measured the evolution of the cell abundances of the heterotrophic bacteria in the samples subjected to different treatments in the three experiments. The abundance increased in most samples. The most significant difference was observed in the M1 experiment, for which the abundance decreased or remained constant after t24 in the VA and VA− treatments and was significantly lower than in the VA+ treatment (*P*<0.05). Growth was thus detected only during the first 24 h and reached high rates. Higher cellular concentrations in the VA+ treatment were also observed during M2, but the patterns (i.e. the increase of abundances) were quite similar between the different treatments. The maximum growth rates had a narrow range during the first 24 h (0.55-0.65 day^−1^) when comparing the different treatments, but there were significantly differences between VA− (0.83 day^−1^), VA (0.49 day^−1^) and VA+ (0.19 day^−1^) between t24 and t48 (*P*<0.05). As for M1 and M2, the bacterial cell abundance remained lower in M3 in VA− compared to VA and VA+. The abundance increased constantly in VA and VA−, while decreasing moderately at the end of the experiment for VA+.

### Effect of treatments on nutrient dynamics

**Fig. 4.**
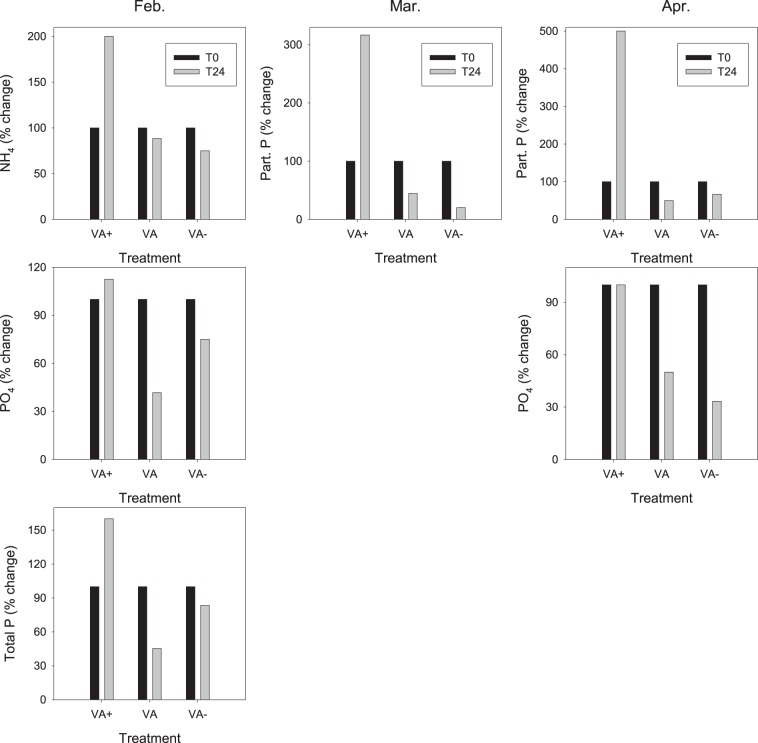
**Increase or decrease of various nutrients from t0 to t24 in the VA+, VA or VA− treatments.** Nutrients are ammonium (NH_4_), particulate and total phosphorus. P in Part. P and Total P refers to orthophosphates (PO_4_).

Different nutrients (total phosphorus, total nitrogen, PO_4_, NH_4_, NO_3_) as well as the dissolved (DOC) or particulate organic matter for the carbon, phosphorus and nitrogen (POC, POP, PON) were analysed at t0, t24 and t48 in the different treatments. For most of the measured parameters, a significant difference was already recorded at t0 between the different treatments so that the potential effect of the increase or decrease in the concentration of viruses was difficult to assess. However, we observed a marked increase after 24 h of incubation for some of the parameters (Fig. 4). Typically, both ammonium and total P and phosphate were significantly higher in the VA+ treatment compared to VA− (*P*<0.05) during the first experiment in February. This was also the case for the particulate phosphorus during M2 and M3. In April (M3), phosphates were also significantly higher in the VA+ treatment compared to both VA and VA− (*P*<0.05).

### Effect of treatments on prokaryotic community structures

**Fig. 5.**
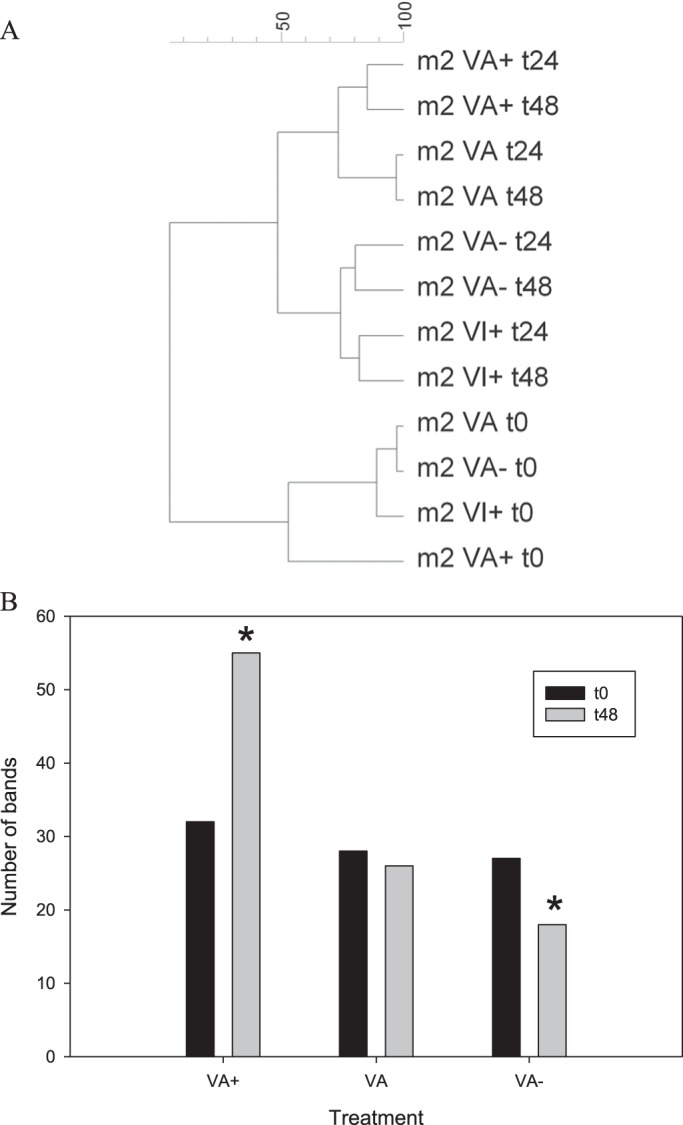
**Changes of the eubacterial community structure.** (A) Clustering analysis for the Eubacteria in the M2 experiment. (B) Evolution of the DGGE band number in the M2 experiment. *P<0.05.

Significant differences in the structure of the bacterial community were revealed by the DGGE analysis after only 24-48 h between the different conditions for the second experiment (*P*<0.05). In March, two clusters separated distinctive treatments at t0 and treatments at t24 – t48 and between VA/VA+ and VA−/VI+ (Fig. 5A). The number of DGGE bands was 29±3 at t0 and 22 to 55 at t48 for all of the treatments. More interestingly, between t0 and t48, we observed a significant (*P*<0.05) increase in band numbers in VA+ (from 32 to 42 bands), while this amount decreased in VA− (from 27 to 18 bands, Fig. 5B). No clear trend was observed among the other experiments carried out during the other periods of the year.

In addition, no significant difference was observed between the treatments for the archaeal community (*P*<0.05).

## DISCUSSION

Weitz et al.’s (2015) recent model highlighted the importance of viruses in shaping the structure of the microbial community and ecosystem function because the model suggests that as the number of viruses increases, the recycling of organic matter increases, as does the primary production and the amount of heterotrophic bacteria, while the transfer to higher trophic levels decreases. Although viruses have received considerable attention during the last three decades, experimental studies dealing with their impact on the nutrient dynamics and community structure still remain relatively scarce, especially for freshwater ecosystems. Investigations in which the viral impact on both nutrient regeneration and diversity has been studied are also very rare. This study began as an extension of a former analysis that was performed in a neighbouring lake of Lake Bourget, Lake Geneva, where we observed that viruses could sustain bacterial diversity during a period in which viral lysis was high (Jacquet et al., 2013). In this former study, we proposed that viruses could control the diversity of heterotrophic bacteria, typically by eliminating the most competitive (e.g. the more active or abundant) members of the prokaryotic community following the “killing the winner” hypothesis of Thingstad and Lingell (1997) and Thingstad (2000), as proposed or demonstrated elsewhere (Fuhrman and Schwalbach, 2003; Jardillier et al., 2005; Hewson and Fuhrman, 2006; Bouvier and del Giorgio, 2007). Interestingly, the study proposed by Jacquet et al. (2013) also revealed that the virus-driven change of the structure of the eubacterial community varied with time, with periods where viruses could indeed be responsible for such a change, but with other periods where flagellates were more likely to be the main biological forcing mechanism for such a change. Our second motivation to perform this study was the recent report of Shelford et al. (2012), which demonstrated that virus-driven nitrogen (e.g. NH_4_) cycling is likely to enhance phytoplankton growth (in particular in the picoplanktonic fraction). These authors revealed the importance of viruses in regenerating N and supporting phytoplankton production. We thus conducted microcosm experiments to examine the effect of viruses on standing stocks of plankton, the structure of the prokaryotic community (using DGGE) and nutrient dynamics at different periods of the year in the oligo-mesotrophic Lake Bourget (France). Lastly, a third motivation was that at several occasions in the past, we observed that virus-mediated cell lysis was important during certain periods of time and likely significantly contributed to both population mortality and changes in the community dynamics and structure in Lake Bourget (Sime-Ngando et al., 2008; Personnic et al., 2009; Thomas et al., 2011; Zhong et al., 2014).

Our experimental treatments, which consisted of a reconstructive approach using incubations of small autotrophic eukaryotes and prokaryotes as well as heterotrophic prokaryotes with low, normal or high viral abundances, allowed us to observe a variable response among the different populations and over time. It was not always possible to significantly increase the viral abundances, which illuminates the limits and drawbacks of this method. However, this expected increase was clear in the M2 treatment, in which a low but significant (*P*<0.05) viral increase for the VLP2 group was observed between t24 and t48, a result that was corroborated by the viral production measurement. VLP2 remained stable and relatively low in both VA and VA− in this period. Although the dynamics of each plankton group changed, there was no clear link between the dynamics of the VLP2 and any of the populations analysed. The same could be said for the nutrients, except for particulate phosphorus, for which significant changes occurred with a significant increase and decrease in the VA+ and VA/VA− treatments, respectively. It was, however, difficult to establish a clear association between the viral and nutrient dynamics, while recent studies have demonstrated the importance of the viruses in producing dissolved organic matter or inorganic nutrients through cell lysis and their use by non-infected populations (Shelford et al., 2012, 2014).

Keeping in mind that our constant experimental conditions were obviously different from natural conditions, an important finding, however, was that the viruses were responsible for a significant change in the structure of the eubacterial community and that the change was rapid (occurring after only 24 to 48 h) in March. When the virus abundance decreased, a loss of bacterial diversity was observed, as already reported for Lake Geneva (Jacquet et al., 2013). The fact that such a virus-driven change was not recorded in all experiments because selective grazing varies throughout the year and the viral impact may also vary with time, probably in response to host and environmental constraints. Such a conclusion was also reported (Jacquet et al., 2013) and demonstrated elsewhere (Thingstad et al., 2014). These results make sense when one knows that grazing is less specific than viruses and mainly controls the abundance of prey, while viral lysis, which is more species or strain specific, controls community diversity (Weinbauer, 2004). No significant changes were found for the structure of the archaeal community in response to viruses, which was also an interesting result. So far, and to the best of our knowledge, only a few studies have been published on the impact of viruses on this community (Winter et al*.*, 2004). These authors could observe, using T-RFLP (a method with a higher resolution than DGGE), that viruses had an effect at the level of operational taxonomic units. Their results highlighted the fact that individual members of the pelagic archaeal and bacterial communities could be affected differently by the presence of virioplankton. Compared to the findings of Winter et al., viruses did not seem to constitute the main factor that impacted in community patterns and changes in Lake Bourget during the period from February to April. Keeping in mind the limits of the DGGE method (which only allows researchers to detect and follow the major and more abundant phylotypes), this result echoes our recent analysis of the structure of the archaeal community for two lakes (including Lake Bourget) at two depths over two years (with one sample taken each month). Indeed, we never observed that viruses were determinant in structuring this community (Berdjeb et al., 2013), while this could be the case for the Eubacteria (Berdjeb et al., 2011). Thingstad and Lignell (1997) suggested that viruses selectively infect the most abundant members of the prokaryotic community and therefore might be a driving force in maintaining prokaryotic diversity. However, a prediction of this theory is that the most abundant viral populations should infect the most abundant host populations. In our case, it is possible that the lack of a viral effect on the archaeal structure could be related to the low abundance of these communities (hosts and specific viruses) compared to the eubacterial and associated phage communities (Comte et al., 2006). The lack of response could also be due to resistance phenomena for the two prokaryotic communities. It is indeed possible that most members (or at least the dominant ones – typically those targeted by the DGGE) were resistant to viral infection. Such resistance (because of a reduction or change in cellular receptor structures or because of lysogeny processes) has been reported (Thomas et al., 2011, 2012 and references therein).

## CONCLUSION

Using an experimental design that consisted of filtering and reconstituting natural waters sampled during different periods of the year, we observed that viruses may exert, on a relatively short time-scale, a significant change in the composition of the prokaryotic community. This was especially true for the eubacteria, likely to be the most numerous and diverse prokaryotes in Lake Bourget. However, we could not demonstrate the relative importance of viruses because nutrient regeneration agents fuel primary producers, as was recently shown for marine ecosystems. This part of the experiment should be reanalysed to observe the possible influence of viruses on resource control and whether viral lysis may supply a significant portion of the nitrogen or phosphorus requirements of phytoplankton and/or non-infected bacteria in freshwater, as exemplified here by the large and deep peri-alpine lakes. The period of investigation (winter versus spring, summer or fall) is likely to be critical and should highlight when viruses may exert an important role and be linked to prokaryotic mortality (Personnic et al., 2009), nutrient release and regeneration (typically when resources are limited) and the dynamics of some specific groups.

## MATERIALS AND METHODS

### Study site

Water samples were collected in Lake Bourget, situated on the western edge of the Alps (45°44′N; 05°51′W; 231 m altitude). It is an elongated and north-south oriented lake (length 18 km; width 3.5 km; area 44×10^6^ m^2^; volume 3.5×10^9^ m^3^; maximum depth 147 m; mean depth 80 m; residence time varying between 8 and 13 years). Lake Bourget is now considered to be oligo-mesotrophic, but it has been characterised by a recurrent bloom of the filamentous cyanobacterium *Plankthotrix rubescens* between 1996 and 2009 that disappeared thereafter in response to lake restoration (Jacquet et al., 2014). More details dealing with the microbial and viral ecology of this lake are available in a variety of studies (Jacquet et al., 2005; Personnic et al., 2009; Sime-Ngando et al., 2008; Thomas et al., 2011; Berdjeb et al., 2011 or again Zhong et al., 2013, 2014). Sampling was conducted between January and May 2013 and was carried out either at various discrete depths between the surface and at a 20 m depth or through a 0-18 m integrated sample. Our experiments were conducted on the 0-18 m integrated water sample as representative of the upper sunlit layer zone. Water was obtained using either a Niskin bottle or an electric pump and appropriate tubing at the reference sampling station of the lake (referred to as point B) located above the deepest part of the ecosystem. All of the samples were placed in sterile polycarbonate bottles and kept in the dark at 4°C until being processed immediately on return to the laboratory (i.e. within 6 h).

### Physico-chemical variables

All nutrients were measured on each date and throughout each experiment, according to standard French normalized protocols. Briefly, the total organic carbon (TOC) was determined after persulphate mineralization heated at 80°C and with IR detection (NF EN 1484 FDT 90-102); dissolved ammonium (NH_4_) was determined by colourimetry using indophenol blue (NF T90-015); dissolved nitrates (NO_3_) were measured by ionic chromatography (NF EN ISO 10304); total nitrogen (TN) was measured by chemiluminescence (NF EN 12260); particulate organic nitrogen (PON) was measured by chromatography with a thermal conductivity detection after combustion/reduction; orthophosphates (PO_4_) were quantified by spectrophotometry, following the molybdenum blue method reported by Murphy and Riley (1962) (NF EN 1189); and total phosphorus (TP) and particulate organic phosphorus (POP) were obtained by the same method, after mineralization (NF EN 1189). During the natural survey, a conductivity-temperature-depth measuring device (CTD SEABIRD SAB 19 Seacat profiler) and a chlorophyll fluorescence Fluoroprobe (BBE Moaldenke, Germany) were used to obtain vertical profiles of the water temperature, conductivity, dissolved oxygen concentration and chlorophyll *a* fluorescence.

### Experimental set-up

**Fig. 6.**
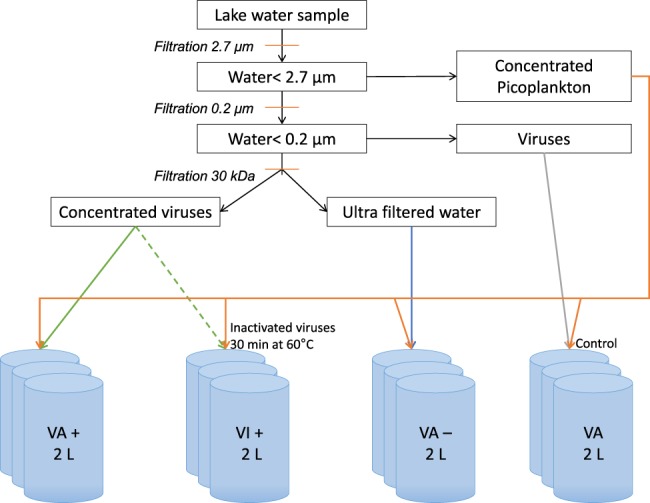
**Diagrammatic representation of the experimental design to examine the effects of viruses on plankton and nutrient dynamics as well as the structure of the prokaryotic community.** VA, viral abundance; VA+, samples enriched with viral fraction, VA−, samples without a viral fraction; VI+, fraction with inactivated viruses.

For each of the three experiments (subsequently referred to as M1, M2 and M3), samplings were carried out in February, March and April. Sixty litres were obtained using a pump and tubing to obtain integrated waters from the surface to a depth of 18 m. For our experiments, the integrated sample underwent a series of filtrations and ultra-filtrations to create different fractions of interest, namely, a cellular concentrate, viruses at a normal abundance, a viral concentrate and ultra-filtered water (Fig. 6). For this purpose, a first filtration was carried out twice on fibreglass membranes (Whatman, porosity 2.7 µm) to remove the metazooplankton, ciliates and majority of heterotrophic flagellates and to keep only the free-living viruses, heterotrophic bacteria, archaea and small autotrophs in the permeate. Note that such pre-filtration resulted in a <5% loss of viral particles or heterotrophic prokaryotic abundance (not shown). The second filtration was performed on a polycarbonate membrane (Nucleopore, Whatman, porosity 0.2 µm), while keeping the fraction under 2.7 µm in suspension, which theoretically can decrease the viral number and concentrate cellular populations over the filter. The permeate <0.2-µm filtered water was subjected to a column ultrafiltration (cutting threshold 30 kDa - Prep/Scale-TFF, Millipore), which allowed the attainment of a virus-free fraction (ultra-filtrated water) on one hand, and a virus-enriched water (viral concentrate) on the other hand. These different fractions were mixed to obtain four experimental conditions (referred to as VA+, VA, VA− and VI+ for virus-enriched, normal, virus-depleted and virus-inhibited fractions, respectively) in 2-litre bottles previously cleaned with a 10% hydrochloric acid solution and rinsed three times with sterile Milli-Q water. The 150-ml concentrated cellular fraction was increased to 2 litre (i.e. 1850 ml were added) with each different solution (i.e. concentrated viruses, ultrafiltrated water or normal viral solution). The 12 bottles (triplicates of the 4 conditions) were incubated for 4 days in a culture room with controlled temperature (18°C) and light (300 µmol quanta m^−2^ s^−1^). These conditions were chosen so that these parameters would not limit population growth.

### Measurements of viral production and the frequency of lysogenic cells

To estimate virus production (VP), we used the dilution technique of Wilhelm et al*.* (2002). Briefly, a 300-ml sample was filtered through 0.2-µm pore size polycarbonate filter. With the vacuum on, 50 ml of the sample was mixed with 250 ml of 0.02-µm ultrafiltered water (virus-free) of the same initial sample, maintaining a final volume of 300 ml. This process resulted in viruses being diluted to approximately 20% of their initial abundance. Triplicates of 100 ml were made and incubated at the *in situ* temperature in the dark. Two-millilitre subsamples were collected at t=0, 6, 12, and 24 h. The VP was determined from the production of new viral particles after the dilution of the initial viral abundance. The VP rates were determined from first-order regressions of viral abundance versus time after correcting for the loss of the bacterial hosts between the experimental samples and the natural lake water community. The viral production was calculated as VP=m×(B/b) (Hewson and Fuhrman, 2007), where m is the slope of the regression line, b the abundance of bacteria after dilution, and B the abundance of bacteria prior to dilution. The viral turnover rates were estimated by dividing the viral abundance by the VP rates.

The fraction of heterotrophic bacteria that were lysogenic was estimated by adding mitomycin C (Paul, 2008) to three replicates of 50-ml samples of lake water at a final concentration of 1 µg ml^−1^. Samples were incubated in the dark at the *in situ* temperature. Two-millilitre subsamples were collected as above and analysed for bacterial and viral abundances. The fraction of lysogenic bacterial cells (FLC) was calculated using the formula of Weinbauer et al. (2003): FLC(%)=100×[(Vmct24−Vct24)/(BS×BAt0)] with Vmct24=abundance of the viruses in the mitomycin treatment after 24-h incubation, Vct24=abundance of the viruses in the control treatment after 24-h incubation, BAt0=initial abundance of the bacteria in the control treatment, and BS=Burst Size (i.e. the number of viruses liberated from a bacterium as a result of lytic infection) using a value of 27 (Zhong et al., 2014).

### Flow cytometry analysis (FCM)

Abundances of virus-like particles (VLP), heterotrophic prokaryotes and picocyanobacteria were measured by flow cytometry (FCM) using a FACSCalibur (Becton Dickinson). Briefly, the VLP and heterotrophic prokaryotes were fixed with 0.2-µm filtered-glutaraldehyde (0.5% final concentration, grade I, Merck) for 30 min in the dark, until being counted, using the same protocol as described in Personnic et al. (2009) and references therein. To analyse the picophytoplankton community dynamics, samples were processed without adding any fixative or dye (all details can be found in Jacquet et al., 2013). All of the cellular abundances were also used to calculate net growth rates (expressed in day^−1^) of the different groups following the equation Ln(A_tf_/A_ti_) with A_tf_=abundance after 24 h compared to A_ti_.

### Bacterial community structure and banding pattern analysis

The analysis of the bacterial or archaeal community structure was assessed using denaturing gradient gel electrophoresis (DGGE). We are aware that DGGE only provides limited information of the dominant groups and about the richness of the natural prokaryotic communities, but in the context of this study, this fingerprinting approach was easy to use and able to reveal major shifts in the dominant groups. After DNA extraction (as described in Berdjeb et al., 2011) and quantification according to the absorbance at 260 nm using NanoDrop ND1000 Spectrophotometer (Thermo Scientific), the extracts of DNA of the sampled community were then stored at −20°C until PCR amplification. PCR reactions were carried out following the PCR cycle described in Berdjeb et al. (2011) for bacteria and Berdjeb et al. (2013) for archaea, using the Eubacteria-specific primer 358-GC, the universal primer 907 rM for the Eubacteria and the primer sets 21F-958R, Parch519 and Arch915 for Archaea. The PCR products were verified by agarose gel electrophoresis. The DGGE approach was performed on PCR fragments, essentially as described in Dorigo et al. (2006), but by using Ingeny PhorU-2 (Ingeny International) and a linear gradient of the denaturants urea and formamide, which increased from 40% at the top of the gel to 80% at the bottom of the gel. Digital images of the gels were obtained using Geldoc (BioRad).

### Data analysis

The DGGE banding patterns were analysed using the GelCompar II software package (Applied Maths, Kor- trijk, Belgium). Briefly, banding patterns were first standardized with a reference pattern included in all gels. Each band was described by its position and its relative intensity in the profiles, which could be described as the ratio between the surface of the peak and the sum of the surfaces for all peaks within the profile. The UPGMA (Unweighted Pair Group Method with Arithmetic Mean) was used as a simple agglomerative hierarchical clustering method, and we analysed the number and presence/absence of the bands for the different treatments at the indicated times.

For the different parameters followed, the differences of the means were tested using two-tailed Student *t*-tests.
